# Effect of rinsing time and surface contamination on the bond strength of silorane-based and dimethacrylate-based composites to enamel

**DOI:** 10.4317/jced.55148

**Published:** 2018-11-01

**Authors:** Farnoosh Fallahzadeh, Mohammad Atai, Shirin Ghasemi, Ailin Mahdkhah

**Affiliations:** 1 Assistant Professor of Operative Dentistry, Faculty of dentistry, Qazvin University of medical science, Qazvin, Iran; 2 Professor of Polymer Science, Iran Polymer and Petrochemical Institute (IPPI), Tehran, Iran; 3 Operative dentistry specialist, Tehran, Iran; 4 Post-graduate Student of Operative Dentistry, Faculty of Dentistry, Qazvin University of Medical Science, Qazvin, Iran

## Abstract

**Background:**

The aim of this study was to assess whether saliva contamination and rinsing time for 15, 30, and 60 seconds, affects the shear bond strength of silorane and methacrylate-based composites to enamel.

**Material and Methods:**

Two light cure resin, P60 (3M ESPE) and Filtek LS Silorane were tested. 120 sound premolars were randomly divided into four groups of 30 teeth based on composite type with or without saliva contamination after etching and rinsing. Each group was further divided into three subgroups according to their rinsing time. Then a cylinder of the composite was bonded to the enamel and Shear bond strength was assessed. To determine the failure mode, the bonded surfaces were then observed under SEM. In addition, the DC of each group was measured at pH levels of 4 and 7 using FTIR spectroscopy. The data were analyzed with one-way ANOVA and post hoc analysis followed by Fisher’s least significant difference.

**Results:**

The bond strength of the non-contaminated methacrylate group was significantly higher than the other groups (*p*< 0.0001). In addition, there was no significant deference between the methacrylate subgroups. In the silorane groups, the shear bond strength was higher in the rinsing time of 15 seconds. Failure pattern was mainly adhesive. The DC of the Methacrylates had no significant difference at pH 4 and pH 7, but was significantly higher than that of siloranes (*p*< 0.0001). While the DC of the siloranes at pH 4 was significantly higher than at pH 7 (*p*< 0.0001).

**Conclusions:**

Saliva contamination in both composites reduces bond strength. Increasing rinsing time in Methacrylates proves ineffective. In non-contaminated siloranes, excessive rinsing time reduced bond strength. The best-recommended rinsing time for both composite is 15 seconds.

** Key words:**Composite resins, silorane composite resin, methacrylates, shear strength.

## Introduction

Composites that are mainly used are methacrylate-based composites, which have some drawbacks such as polymerization shrinkage that leads to stress at the tooth-composite interface (1). A new monomer system called silorane has been developed to reduce shrinkage and internal stresses resulting from polymerization (2,3). The silorane matrix is formed by the cationic ring-opening polymerization of the silorane monomers, which can reduce the polymerization shrinkage to less than 1.0 vol.% (4,5). Comparing with the shrinkage of 1.82% to 2.19% for methacrylate-based resin composites (6). This, results in a significant decrease in microleakage and improved marginal adaptation (7,8). Siloxane provides the composite with hydrophobicity, which brings about a reduction in water absorption and colour change and an increase in the physical strength of the composite material and hydrolytic stability (9). In addition, Siloranes have demonstrated variable marginal leakage (10), similar photo polymerization efficiency (11), low toxicity (12) and comparable physical properties (11), compared to methacrylate composites.

Etching with phosphoric acid results in the deposition of calcium and phosphate on tooth surface that interferes with successful bonding. Thus, the deposits should be rinsed from the surface with water (13). The effective rinsing time is when the etching material and the deposit have been removed, and it should be as short as possible to reduce the possibility of contamination with saliva (13). However, most studies reported that etchants were removed with much shorter rinse times than recommended instructions of manufacturers (14-16). Additionally, rinsing time can influence the chemical nature of the surface, including surface pH. By increasing the rinsing time, pH increases in such a way that this increase can influence the polymerization of the composite (4,17). In the case of cationic polymerization (based on silorane), an acidic environment can stimulate polymerization, but its procedure is slower than the anionic type4.

After etching, adhesive agents penetrate into the pores and create the micromechanical bonding. Hence, any contamination of the prepared surface with saliva, gingival crevicular fluid, or blood should be avoided to achieve a proper bond between the composite and the tooth. If contamination occurs, pores will be filled with the contaminants instead of adhesive and micromechanical retention will lower (18,19).

With the advent of silorane-based composites, a few studies were conducted on the appropriate clinical conditions, physical properties and polymerization characteristics for the successful performance of these composites. However, some questions remain about the behavior of these materials in different conditions of bonding preparation and contamination.

The aims of this study were: 1) to evaluate the effect of rinsing time and surface contamination with saliva on bond strength of low-shrinkage silorane-based resin composites and conventional methacrylate-based resin composites. 2) To explore the effect of surface pH on the DC of methacrylate- and silorane-based composites, and 3) to assess the mode of bond failure in these composites.

## Material and Methods

This *in-vitro* study was carried out on 120 freshly extracted sound human premolars. The teeth were cleaned from soft tissue remnants and debris with hand instruments and were stored in distilled water containing 0.1% thymol at 4 °C until they were used (within three months of extraction). Twenty-four hours before the experimental procedure, the teeth were immersed in deionized water at a temperature of 23±2 ºC. To prepare the samples, the roots of teeth were sectioned up to 2 mm below the cemento-enamel junction with a separating disk, and then the lingual side of the crowns was sectioned with a low-speed diamond saw (IsoMet, Buehler, Illinois, USA) under a water coolant so that the cutting surface could be completely flat. The buccal surface of each tooth was grounded using the Soflex’s (3M ESPE) coarse, medium, and fine grit polishing disks, in that order. Each disk was used for five teeth and then was replaced with a new one.  Table 1 presents the characteristics of the materials used in the present study.

**Table 1:**
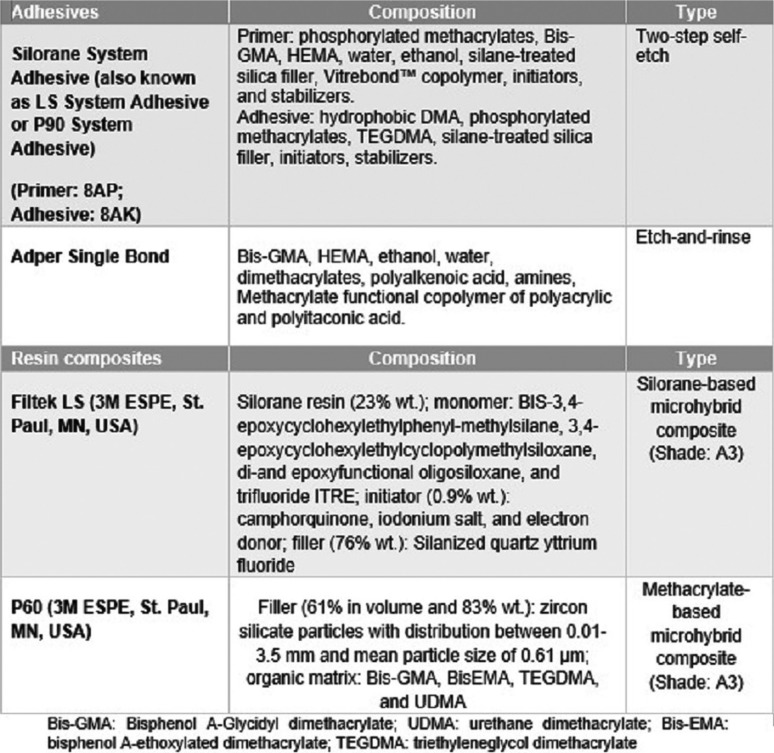
Materials used in the study (3M ESPE, St. Paul, MN, USA).

**Figure 1:**
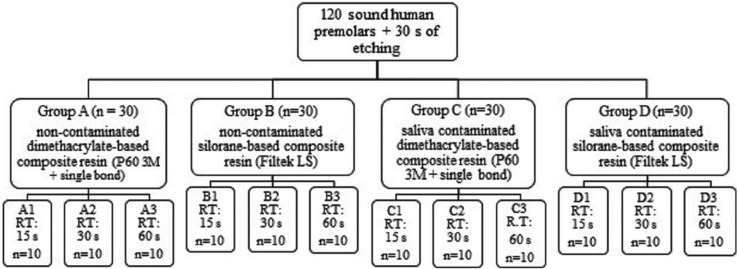
Classifying samples based on adhesive system, contamination type, and rinsing time (15 s, 30 s, 60 s) (RT: rinsing time).

Then, the samples were randomly divided into four groups of 30 teeth each based on the composite resin type and contamination. Each group was further divided into three subgroups of 10 teeth according to the rinsing time. Classification of the groups is shown in Figure 1. In each subgroup, the teeth were etched with 37% phosphoric acid gel (Total Etch, Kerr; Danbury, CA, USA) for 30 seconds. In non-contaminated groups, after etching and rinsing under one of the three rinsing times (i.e., 15, 30, or 60 seconds), a gentle oil-free airstream was directed at the samples at a distance of 1 cm for 5 seconds for drying purposes. The same procedure was carried out for the contaminated groups, with the only difference being that after etching and rinsing, fresh saliva was applied to the etched surfaces. For this purpose, unstimulated saliva was collected from one healthy female volunteer who was informed of research protocols and had signed a written consent. She had been instructed to brush her teeth and avoid eating for one hour before the treatment. Saliva collection took place in one session immediately prior to the bonding procedure. Excessive saliva was removed using cotton rolls.

In methacrylate-based groups, single bond (3M ESPE) and P60 (3M ESPE) were used for bonding application according to the instructions of the manufacturer. Two layers of single bond were applied using a micro-brush and were thinned with a gentle airstream. Then, the prepared surface was light cured using Optilux 501 (SDS Kerr, Danbury, CA, USA) for 20 seconds. The intensity of the curing device that ranged from 500 to 520 mW/cm2 was measured periodically using a radiometer (Demetron/Kerr Corp, Orange, CA, USA). In silorane-based groups, primer was applied with a micro-brush followed by gentle air dispersion and 10 seconds of light curing. Then adhesive was applied with a micro-brush, followed by gentle air dispersion and 10 seconds of light curing.

In order to test the microshear bond strength, the protocol described by Shimada *et al.* (20) (2002) was used. A silicone tube with an internal diameter of 0.75 mm was cut into 1 mm in length using a gage and blade to ensure parallel ends (TGY-030, Small Parts Inc., Miami Lakes, FL, USA). The composite was filled into the silicone tubes and inspected for any defect within the composite. The composite-filled tubes were then placed perpendicular on the buccal tooth surface and were light cured for 40 seconds. Finally, a scalpel (No. 11) was used to cut away the tube. If the cured composite showed any gap formation, bubble inclusion, or any other defect, the respective sample was excluded from the study. After the bonding procedure, all samples were placed in distilled water in an incubator (Dorsa, Tehran, Iran) at 37 °C for 24 hours.

-Microshear bond strength test

**Figure 2:**
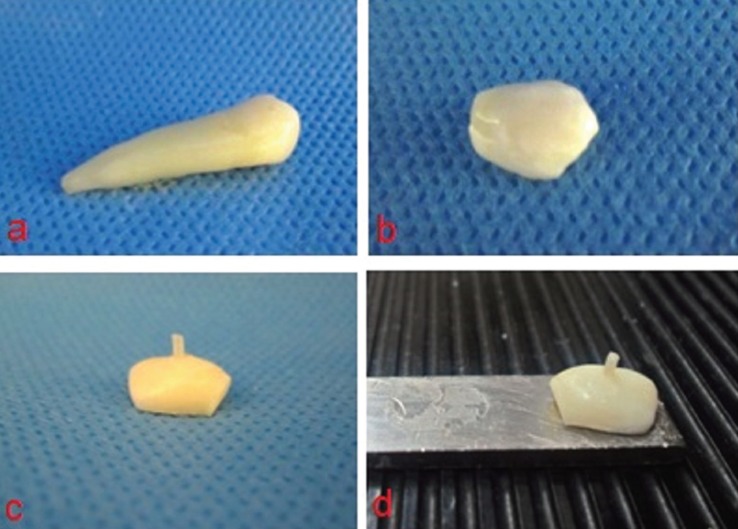
Sample preparation steps.

After 24 hours of storage in water, the samples were attached to the testing device using cyanoacrylate adhesive (Alpha Glue, Razi Chemical Co., Iran), (Fig. 2). The shear bond strength was evaluated with a universal testing machine (STM-20, Santam, Iran). To ensure close contact with half of the composite/enamel junction, the ligature wire was carefully looped around the composite cylinder bonded on tooth enamel surface. The wire was then held flush against the resin/enamel interface. The wire loop and the center of the load cell were aligned as straight as possible to ensure that the desired orientation in shear stress was maintained. A shear force was subsequently applied to each sample at a crosshead speed of 1.0 mm/min, ( Table 2). The micro shear bond strength was calculated by dividing the maximum load at failure by the cross-sectional surface area of the bonded resin surface. If a spontaneous debonding occurred before bond strength testing, the sample was excluded from the study. All the procedures performed with the same operator.

**Table 2:**
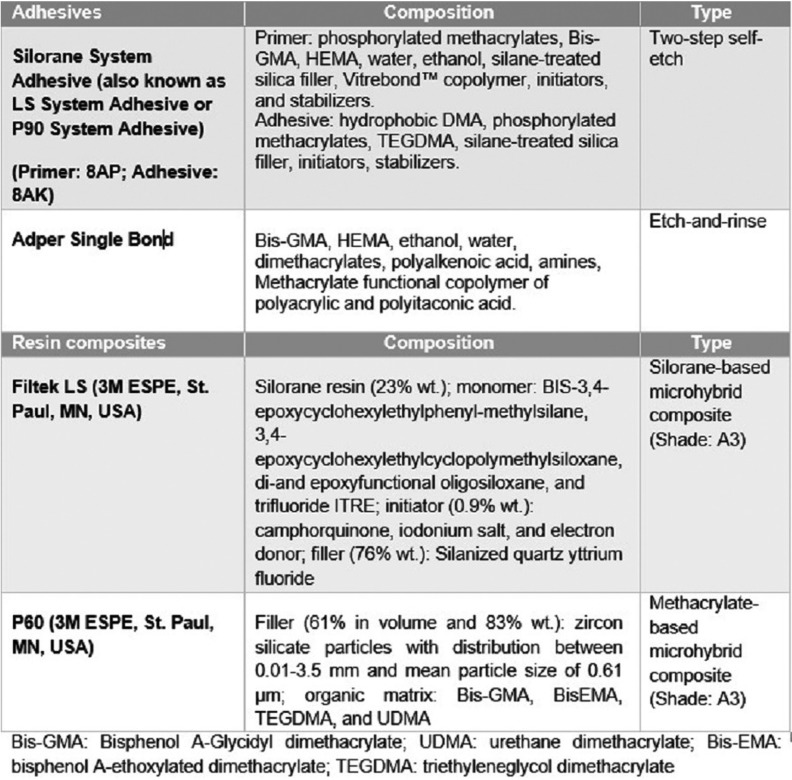
Samples placed within the fixed jaw of the universal testing machine.

-Scanning electron microscopic evaluation

Scanning electron microscope (SEM) was used for determining the mode of bond failure. From each subgroup, five randomly selected debonded teeth were mounted on stubs, sputter coated with gold (SEM coating unit 5100, Polaron instruments Inc., Agawan, MN, USA) and examined under a SEM model VEGA II (TESCAN-LMU, Brno, Czech Republic) at ×110 magnification to determine failure modes. Based on the SEM micrographs the failure modes were classified as adhesive, cohesive (in enamel or composite), or mixed.

-Fourier-transform infrared spectroscopy (FTIR)

To evaluate the effect of surface pH on the DC of the composites, for each composite type, two subgroups of 10 teeth were defined for pH 4 and pH 7. First, a small amount of uncured composite paste was sandwiched between two polyethylene strips soaked in water with a pH of 4 or 7 and pressed between two glass slides to obtain a thin film. The infrared spectra of uncured samples were collected using an FTIR spectrometer (Equinox 55, Bruker, Germany) at a resolution of 4 cm-1 and a spectral range of 4000-400 cm-1. Later, the samples were irradiated for 40 seconds according to the curing protocol discussed above and their FTIR spectra were obtained.

The DC% was measured according to the following equations, which is based on the decrease in the absorption intensity bond ratios before and after light curing. the absorbance intensity was measured of aliphatic double bond at 1637 cm-1 and epoxy rings (C-O-C bond) at 884 cm-1 for methacrylate and silorane based composites, respectively. The absorbance peaks at 1608 cm-1 for methacrylate aromatic double bond and at 458 cm-1 for silorane based composite, were considered as internal reference.

Methacrylate DC = [1- (peak 1637/peak 1608 after curing)/(peak 1637/peak 1608 before curing)] ×100

Silorane DC = [1- (peak 884/peak 458 after curing)/(Peak 884/peak 458 before curing)] × 100.

The results were analyzed using one-way ANOVA and post hoc analysis followed by Fisher’s least significant difference (LSD) (α = 0.05) on SPSS 22 (IBM, Chicago, IL. USA).

## Results

-Microshear bond strength test

 Table 3 presents the mean, standard deviation, and maximum and minimum values of micro shear bond strength in the study groups. The results of one-way ANOVA showed no significant difference in bond strength during the rinsing periods of 15, 30, and 60 seconds for both non-contaminated and contaminated methacrylate groups (*p* > 0.05). As for the non-contaminated silorane group, bond strength values at the rinsing times of 15 and 30 seconds were not significantly different (*p*> 0.05), but they were significantly higher than bond strength at the rinsing time of 60 seconds. In the contaminated silorane group, bond strength value at the rinsing time of 15 seconds was significantly higher than the values at 30 and 60 seconds. In addition, the bond strength of the samples at the rinsing time of 30 seconds was significantly higher than at 60 seconds (*p* < 0.05). In addition, at the rinsing times of 15, 30, and 60 seconds, the bond strength of the non-contaminated methacrylate group was significantly higher than that of all the other groups (*p* < 0.0001). In this respect, the contaminated methacrylate group was next, followed by the non-contaminated silorane group and the contaminated silorane group (*p* < 0.0001).

**Table 3:**
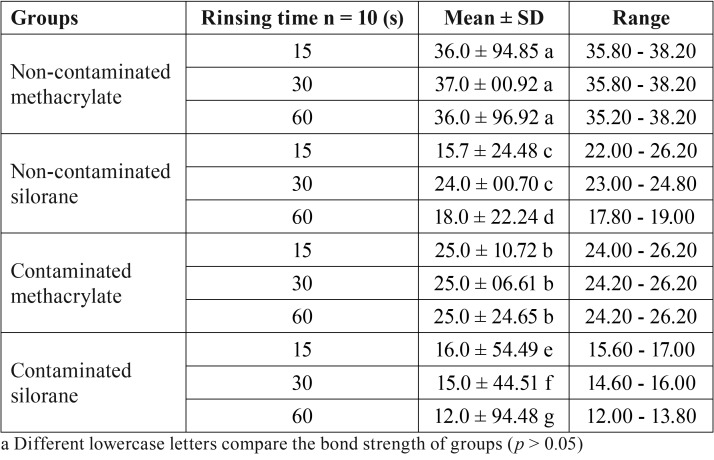
Mean, standard deviation, and maximum and minimum values of micro shear bond strength (Mpa).

-SEM evaluation

SEM examination of the 60 selected samples showed that the most frequent failure mode (90%) was adhesive failure. 10% of the specimens presented mixed failure mode, and no cohesive failure was detected. No relationship was found between failure mode and micro shear bond strength and between failure mode and composite type.

-FTIR evaluation

The FTIR data obtained are presented in  Table 4. As can be seen, at both pH 4 and pH 7, the DC of the methacrylate composites is significantly higher than the DC of silorane-based composites (*p* < 0.0001). It can also be observed that for the methacrylate composites, there was no significant difference between pH 4 and pH 7 (*p* > 0.05), while for the silorane composites, the DC at pH 4 was significantly higher than at pH 7 (*p* < 0.0001).

**Table 4:**
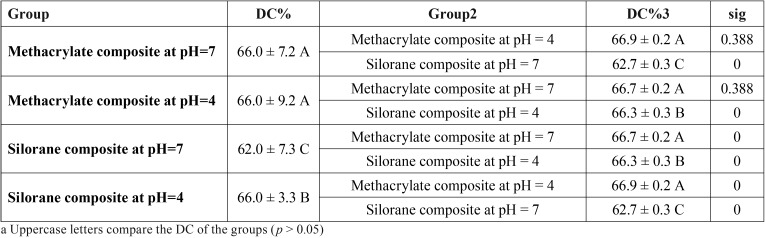
The DC of the methacrylate and silorane composites at pH levels of 4 and 7.

## Discussion

This study analyzed the effect of saliva contamination on the shear bond strength of silorane- and Dimethacrylate-based composite resins at different rinsing time. Also evaluated the effect of surface pH on the DC of both composites.

Overall, methacrylate composite showed a higher bond strength than silorane composite at the three rinsing times of interest on both groups (i.e. contaminated and non-contaminated). In other words, although the silorane system is based on cationic polymerization (that occurs by cationic ring opening), which can reduce the polymerization shrinkage compared with methacrylate-based resins (21), the silorane-based composite did not lead to a higher bond strength. It is important to observe that the FTIR results revealed that the degree of the polymerization of methacrylate composites is generally higher than that of silorane composites, in line with the results reported by Lien and Vandewalle (22) (2010) and Knezović *et al.* (23) (2003). This difference might explain the higher bond strength of methacrylates than that of the silorane group. Additionally, the low polymerization shrinkage of a composite does not always indicate decrease in shrinkage stress on bonding interface (21). Boaro, *et al.* (24) have suggested that the silorane-based resin forms a low-viscosity layer and may induce shrinkage stress similar to that produced by methacrylate resins. During the polymerization of elastic-viscous materials, the viscoelastic behavior changes and can develop strains. Also, the low initial flow presented by the resin can limit the flow of silorane and increase the stress (25). The other aspect that may explain the results of the present study is the hybrid layer formation of these composites. The primer agent of the silorane restorative system presents different curing method from Single Bond. The silorane primer agent is first light cured, therefore, in contrast with the conventional etching adhesive systems, where the hybrid layer formation is determined by the combination of primer and bonding agent, only the primer agent creates the hybrid layer and create a weak bonding interaction between the two substrates that may compromise the bond strength (25).

Moreover, in both methacrylate and silorane composites, saliva contamination led to reduced bond strength. Possibly due to the ability of saliva to penetrate and block the pores created by etching. As for silorane, the reduction of bond in the contaminated group may be related to the fact that saliva contributes to an increase in the pH and overcomes the acidic nature of the silorane composite and the primer26. Like our study, Taskonak, *et al.* (26) (2002) studied the effect of saliva contamination on the shear bond strength of three one-bottle adhesives (i.e., syntac single component, prime & bond NT, and gluma one bondand), which were dimethacrylate-based composite resins and showed that saliva contamination leads to reduced shear bond strength. Jiang, *et al.* (27) (2010) examined the effect of saliva contamination on the bond strength of four self-etching adhesives (Clearfil SE Bond + Clearfil AP-X; Xeno III + Ceram X; Frog + Ice; FL Bond II + Beautifil II). They showed that contamination with saliva dramatically reduces bond strength. In a study by Guo, *et al.* (28) (2017), the effect of saliva contamination on the bond strength of two commercial methacrylate resin composites (AP-X and P60) was examined. It was shown that saliva contamination reduces bond strength. Munaga, *et al.* (29) (2014) found that saliva contamination significantly decreases the strength of the shear bond between the silorane-based P90 system adhesive and dentin.

Although, this *in vitro* test showed rinsing time (15, 30 and 60 s) had no effect on the bond strength of methacrylate composites, but in the silorane composite groups, increasing the rinsing time, led to a decrease in bond strength. This could be due to the DC of these composites after light curing. According to the FTIR results, change in the pH does not affect the DC of methacrylate composites, implying that any increase in the pH emanating from an increase in rinsing time dose not have a significant impact on the bond strength of the methacrylate composites. In addition, the results of FTIR showed an increase in the DC of silorane composites at low pHs. Thus, it can be deduced that by increasing the rinsing time to 60 seconds, surface pH increases and the DC reduces, bringing about reduced bond strength. This finding concurs to some extent with a study by. Bates *et al.* (15) (1982) which reported no significant difference in tensile bond strengths of specimens rinsed for 5, 10, or 30 seconds after a 60-second enamel etch. Also, Turner *et al.* (13) (1987) concluded that. Mean counts per minute (CPM) for radiolabelled gel etchants reduced from 56,223 CPM at 0-second rinses to 28 CPM at 5-second rinses. Interestingly, no statistically significant reduction was observed in residual radioactivity with 5- to 60- second rinsing. Furthermore, Mixson *et al.* (16) (1989) compared the shear bond strength obtained by varying rinse volumes (0, 2, 5, 10, 15, and 25 ml of water) and different air and water pressures (20/10, 20/40, 40/10, and 40/40 psi). A significant difference was found between the 0 ml of water volume rinse group with the other volume groups (*p* < 0.05), and the difference between the other groups did not reach significance. They found that any volume greater than 2 ml, which is the threshold for removing calcium and phosphate deposits, did not dramatically increase bond strength and that water pressure did not affect bond strength. However, Turner *et al.* (13) (1987) reported that the right time for rinsing the liquid etchant was 15 seconds and it was 30 seconds for rinsing the etch gel. The discrepancy between the findings of Turner *et al.*’s study and our observations may be attributed to the different etchants used.

Moreover, an increase in the rinsing time does not influence the bond strength in the contaminated methacrylate group. This can indicate that if the rinsing time is increased (within the range examined in the present study), the deposits of saliva cannot be removed. In the contaminated silorane composite group, the three rinsing times were significantly different in terms of shear bond strength and the 15 seconds rinsing time subgroup had significantly higher values than other subgroups. This could be put down to the combined effect of saliva and rinsing time on an increase in pH level, which prevents the stimulation of polymerization and reduces bond strength. attributed to the fact that the Cationic polymerization is accelerated in the lower Ph. Level4.

Overall, the present study found 15 seconds to be the preferred rinsing time for both methacrylate- and silorane-based composites owing to the effect of pH and acidity on polymerization and in turn on the bond strength of silorane and considering the fact that shorter rinsing periods lead to less recontamination (13,17). Although, rinsing time of less than 15 seconds is suggested to be evaluated in future studies.

## Conclusions

Within the limitations of this study, it was observed that contamination with saliva in both methacrylate- and silorane-based composites generally reduces bond strength, meaning that both composites are sensitive to treatment. It was also shown that increasing the rinsing time is ineffective in the case of methacrylate composites (both contaminated and non-contaminated). However, for contaminated and non-contaminated silorane, an increase in the rinsing time reduces bond strength. Therefore, for both composites, 15 seconds can be regarded as the optimum rinsing time compared to 30 and 60 seconds.
